# Increase of pertussis cases in the Vallès region, Catalonia, Spain, September 2023 to April 2024

**DOI:** 10.2807/1560-7917.ES.2024.29.24.2400332

**Published:** 2024-06-13

**Authors:** Violeta Poltorak, Alba Cabré-Riera, Ferran Martínez-Botías, Eva Borràs López, Laura Clotet Romero, Maria Rosa Sala Farré, Mireia Jané Checa, Working Group for surveillance of pertussis in Vallès

**Affiliations:** 1 Department of Preventive Medicine, Hospital Universitari de Bellvitge, Bellvitge Biomedical Research Institute (IDIBELL), L'Hospitalet de Llobregat, Barcelona, Spain; 2 Public Health Agency of Catalonia, Generalitat de Catalunya, Barcelona, Spain; 3 CIBER Epidemiologia y Salud Pública (CIBERESP), Carlos III Health Institute, 28029, Madrid, Spain; 4 Department of Medicine, University of Barcelona, Barcelona, Spain; 5 The members of the Working Group for surveillance of pertussis in Vallès are listed under Collaborators

**Keywords:** Spain, air-borne infections, bacterial infections, pertussis, outbreaks, surveillance, vaccines and immunisation, epidemiology

## Abstract

We describe a pertussis outbreak in the Vallès region of Catalonia, from September 2023 to April 2024. Incidence was high in children aged 10–14 years compared with previous outbreaks. Limited impact in newborns could be explained by the high vaccination coverage during pregnancy and at 11 months of age in 2022, at 85% and 94.1 %, respectively. A third booster vaccine dose during preadolescence should be considered and vaccination coverage in pregnant women be improved to control future outbreaks.

Pertussis or whooping cough is a contagious respiratory disease caused by *Bordetella pertussis*. Pertussis is a worldwide distributed infection with a cyclic pattern, with peaks every 3–5 years [1]. We describe an outbreak of pertussis in the Vallès region (population 1,342,465), in the northern metropolitan area of Barcelona in Catalonia, from September 2023 to April 2024 mainly affecting 10–14-year-olds.

## Pertussis surveillance in Catalonia

Pertussis is a statutorily notifiable disease in Spain upon clinical suspicion (i.e. cases that meet clinical criteria or confirmation of illness) [2]. Clinical criteria of whooping cough include cough with one of the following symptoms: whooping cough, crowing or high-pitched whoop, vomit, or apnoea. Confirmation is based on detection of *B. pertussis* from a clinical specimen, by real-time PCR or by an epidemiological link to a laboratory-confirmed case. Cases residing in the Vallès region are notified to the Epidemiological Surveillance Service of Vallès (ESSVV). In the notification, sociodemographic data (age, sex, place of residence, school or workplace), clinical symptoms, vaccine history, and epidemiological information (previous contact with suspected or confirmed cases) are recorded on a standardised questionnaire. Contact tracing is done for all cases.

## Epidemiological situation after September 2023

**Figure 1:**
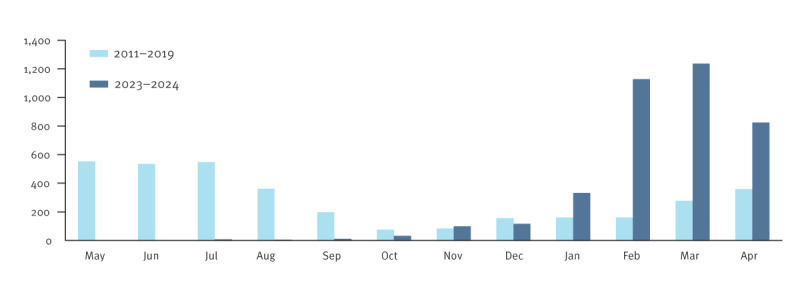
Temporal distribution of pertussis cases in Vallès region, Catalonia, Spain, January 2011–December 2019 (n=3,465) and September 2023–April 2024 (n = 3,775)

**Figure 2:**
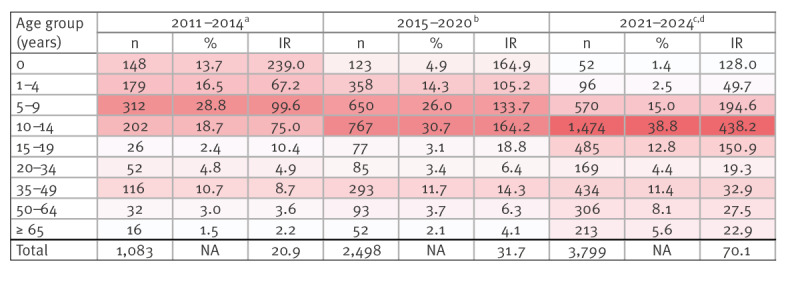
Age distribution of pertussis cases in Vallès region, Catalonia, Spain, 2011–2024 (n = 7,380)

IR: incidence rate; NA: not applicable.

^a^ Period 2011–2014: before vaccination program in pregnant women was implemented in Catalonia.

^b^ Period 2015–2020: vaccination program in pregnant women widely implemented and COVID-19 pandemic started.

^c^ Period 2021–2024: post COVID-19 pandemic and vaccination program in pregnant women implemented.

^d^ Only three cases were confirmed 2021–2022.

From September 2023 to April 2024, 3,775 pertussis cases were confirmed in Vallès region, corresponding to an incidence rate of 281.2 cases per 100,000 population during this period. Vallès region represents 17% of the Catalan population, however, the confirmed cases constituted 50.3% of the total 7,507 Catalan cases during this period [3]. The case numbers were higher than those reported before the COVID-19 pandemic, as in the other regions of Catalonia (Figure 1) [4]. Previous outbreaks in Catalonia were in 2011 [5], 2015 and 2017, when 421, 867 and 504 confirmed cases were notified, respectively, but during the COVID-19 pandemic years, the notifications and subsequent case confirmations decreased (Figure 2). Of the 3,775 cases, 52.3% were female. The seasonal pattern changed in the current outbreak: the number of cases started to increase in January, with a peak of 1,127 and 1,235 cases in February and March, respectively. Usually, the highest case numbers are recorded in May and June (Figure 1). The incidence in the ongoing outbreak was higher in children and adolescents aged 10–14 years (1,772.2 cases per 100,000 population) compared with previous outbreaks, with fewer cases in children aged < 1 year (527.7 cases per 100,000 population). There has been a shift in the age distribution from 2011 to 2024 (Figure 2). From 2011 to 2015, 13.7% of cases were younger than 1 year and 18.7% were aged 10–14 years. However, between 2021 and 2024, 1.4% of cases were younger than 1 year and 38.8% were 10–14 years-old. In the current outbreak, few cases were younger than 1 year (n = 51, 1.4%).

Of the 51 cases younger than 1 year, 35 were fully vaccinated according to the recommended schedule, seven were not vaccinated or only partially vaccinated and nine were below vaccination age (i.e. aged < 2 months). Mothers of 35 cases were vaccinated during pregnancy, on average during week 31 of gestation (range: 27–32).

So far, no fatalities were reported. Of the 3,775 cases, 26 were hospitalised, 12 of them were younger than 1 year of age; seven of the 12 hospitalised children needed treatment in intensive care unit (ICU) and mothers of eight children were vaccinated during pregnancy, four of whom needed ICU treatment. Of the 13 hospitalised adults, 11 were aged > 65 years. No adults needed ICU treatment and none of the hospitalised adults was previously vaccinated.

## Public health measures to control the outbreak

Treatment and isolation were recommended for all confirmed cases. According to the Catalan protocol in force until April 2024 [2], chemoprophylaxis was recommended to contacts of confirmed cases that were children aged < 1 year, women in the last month of pregnancy or persons working for or with them. In addition to chemoprophylaxis, vaccination was recommended for persons living or working with children aged < 1 year or persons working with pregnant women if vaccinated more than 5 years earlier or not vaccinated. Other actions taken were training (i.e. webinars) for health professionals to increase clinical suspicion and diagnostic sensitivity, improve communication circuits between public health and healthcare professionals, give accurate information to family of cases to promptly consult if another family member started with whooping cough compatible symptoms and recommendation of opportunistic vaccination to any case’s contacts aged ≤ 18 that were partially vaccinated or not vaccinated.

## Discussion

During the last 8 months, there has been a considerable increase in the number of cases of whooping cough in the Vallès region. Unlike in previous years, the incidence peaked in February. Similar situations have been described in other European countries and in China [6,7]. This off-season pertussis outbreak might be related to the impact of some of the public health measures during the COVID-19 pandemic, which affected the epidemiology of pertussis and other infectious diseases [8].

Catalonia began vaccinating against pertussis in 1965, with several changes introduced since then. Children were given five vaccination doses from 1999 onwards, the last dose was of a diphtheria, tetanus, acellular pertussis (DTaP)-type [9]. In 2001, primary vaccination with a diphtheria, tetanus, whole-cell pertussis (DTwP) vaccine was replaced with an acellular vaccine (DTaP). In 2016, the primary vaccination schedule with DTaP-*Hemophilus influenazae* type b (Hib)-inactivate p poliomyelitis (IPV)-hepatitis B (HepB) (hexavalent) vaccine changed from three doses to two doses given at the age of 2 and 4 months, and children were given two booster doses, the first, hexavalent, at the age of 11 months and the second, DTaP-PI, at the age of 6 years. In 2022, 94% of 11-month-old children were vaccinated with a booster vaccine dose of hexavalent vaccine and 78.4% of 6-year-olds with dTap [10].

In 2014, vaccination of pregnant women during the gestation week 27–36 started. Catalonia, together with Ireland, were the first European countries starting to vaccinate pregnant women against pertussis [11]. In 2022, 85% of pregnant women were vaccinated. Vaccination with dTap is recommended in each pregnancy, even if the woman has a previous history of pertussis or vaccination, and even if consecutive pregnancies occur within 12 months [12]. In some studies, the ideal timing for dTap vaccination during pregnancy is as close as possible to gestation week 27 [13-15].

In contrast to previous years, the present outbreak mainly affected children and adolescents aged 10–14 years (39.1% of cases), vaccinated with five doses, and receiving the last dose dTap at 6 years of age. This outbreak occurring despite the high immunisation rates against whooping cough in children might be explained by waning immunity against pertussis [16]. Relatively few cases (n = 51) occurred in infants under 1 year of age likely because of the high pertussis vaccination rate among pregnant and paediatric population. Moreover, a progressive reduction of cases in the < 1 year-old age group has been seen in Catalonia and in other countries with establish pregnant vaccination programmes [17,18].

## Conclusion

The pertussis epidemiological situation in the Vallès region, Catalonia, compared with previous outbreaks, shows that the incidence has shifted from children < 1 year to 10–14-year-olds. Importantly, the severity has decreased among small children, reducing the number of hospitalisations in infants aged < 1 year. This change reflects the benefits of the vaccination programme among pregnant women in Catalonia. Nevertheless, the vaccination coverage in pregnant women could be improved and shortening the recommended time window of vaccination of pregnant women might further protect newborns, especially preterm babies. Additionally, the systematic paediatric vaccination programme might be revised to increase vaccination coverages at 6 years of age and to assess the possibility of including a booster vaccine dose during preadolescence, preferably at 10–11 years, which could help control future outbreaks.
